# Changing Incidence and Survival of Primary Central Nervous System Lymphoma in Australia: A 33-Year National Population-Based Study

**DOI:** 10.3390/cancers13030403

**Published:** 2021-01-22

**Authors:** Alexandra L. Farrall, Justine R. Smith

**Affiliations:** College of Medicine & Public Health, Flinders University, Bedford Park, SA 5042, Australia; alix.farrall@flinders.edu.au

**Keywords:** central nervous system, Lymphoma, diffuse large B-cell lymphoma, incidence, survival

## Abstract

**Simple Summary:**

In Australia, all cancers are recorded in the Australian Cancer Database. We used pathological and anatomical classification codes to identify cases of brain lymphoma—termed primary central nervous system lymphoma or PCNSL—that had occurred in Australia during the past four decades. This allowed us to calculate the incidence of PCNSL, as well as the overall survival after diagnosis with this lymphoma. PCNSL is rare, recently affecting approximately 4 per one million adult Australians per year. However, the number of people diagnosed with this cancer is increasing. Survival of people diagnosed with PCNSL has been improving, but is still low, with only 33% of people alive at 5 years after receiving this diagnosis. As the first survey of the incidence of PCSNL in Australia and the survival of Australians diagnosed with this rare cancer, our research supports current efforts to understand risk factors and improve outcomes.

**Abstract:**

Primary central nervous system lymphoma (PCNSL) is a rare brain cancer that remains challenging to study. Epidemiology of PCNSL in the Australian population, which is racially and ethnically diverse, has not been examined previously. Using ICD-O-3.1 Morphology and Topography Codes to identify cases, we analyzed complete datasets from the comprehensive Australian Cancer Database (1982–2014, adults aged ≥ 20 years) to establish incidence rates and trends of PCNSL, and to define survival outcomes of individuals diagnosed with PCNSL, including the predominant diffuse large B-cell lymphoma (DLBCL) type. Age-standardized incidence of PCNSL increased by an average annual 6.8% percent over the study period, with current incidence of 0.43 (95% confidence interval, 0.41–0.46) per 100,000 person-years, in comparison to 21.89 (21.41–22.38) per 100,000 person-years for non-CNS lymphoma. Increase in incidence was characterized by an acute rise between 1996 and 1999, was more pronounced with increasing age, and was driven by increasing incidence of DLBCL. Overall survival for persons diagnosed with PCNSL improved significantly across the study period, with 5-year survival probability increasing from 0.21 (95% confidence interval, 0.16–0.26) to 0.33 (0.30–0.36), and median survival increasing from 318 to 600 days, between 1982–1999 and 2000–2014. Increase in survival was significantly higher for persons with DLBCL versus non-DLBCL PCNSL, but substantially lower than that for persons with non-CNS lymphoma, who had a 5-year survival probability of 0.62 (0.62–0.62) and a median survival of 3388 days in 2000–2014. This study links increasing incidence of PCNSL in Australia to increasing incidence of DLCBL, including in younger adults, and highlights the improving, but low, survival outcome of this cancer.

## 1. Introduction

Primary central nervous system lymphoma (PCNSL) is a rare cancer that exclusively or concomitantly involves the brain, spinal cord, cranial nerves, eyes, and meninges [1]. This cancer is not restricted by region, or racial or ethnic background, and typically presents during the fifth or sixth decade of life. Primary CNS lymphoma accounts for 2% to 3% of all brain tumors [2,3], and non-Hodgkin diffuse large B-cell lymphoma (DLBCL) is the most common histopathological type [1]. Despite considerable advances in the treatments for lymphoma overall, clinical outcomes of PCNSL remain poor; recent reviews have estimated the 5-year survival for immunocompetent individuals at approximately 30% [1,4]. Congenital immunodeficiencies and other immunocompromised states—associated with, for example, human immunodeficiency virus (HIV) infection, immunomodulatory drug therapy and chemotherapy—increase the risk of PCNSL and confer even lower cancer survival rates [5,6,7,8].

Current understanding of PCNSL incidence rates and survival outcomes is based primarily on cohort data from clinical trials, including prospective intention-to-treat studies. Given the limitations inherent in selection criteria and other study parameters, however, such data may not reflect real-world experience. Population-based studies offer the most accurate representation of the epidemiology of PCNSL in the general population. A small number of population-based surveys have been published, including from countries in Asia, Western Europe, and North America; the annual incidence of PCNSL across these regions during time periods spanning 1975 to 2015 ranges from 1 to 4 per million persons, and several studies suggest the incidence is increasing [2,9,10,11,12,13].

Incidence rates and survival outcomes of PCNSL in the Australian population have not been reported previously. We analyzed data lodged in the Australian Cancer Database for all cases of PCNSL that were diagnosed between 1982 and 2014. Incidence rates of PCNSL and survival outcomes for individuals with this cancer were calculated and compared with those of non-CNS lymphomas and non-hematological primary CNS cancers. The population of Australia is largely migrant-based, and today almost 50% of Australians are born overseas or have at least one parent who was born overseas [14]. Thus, a study of PCNSL in Australia contributes epidemiological information for a diverse population with multiple racial and ethnic backgrounds, in addition to providing a new regional perspective.

## 2. Materials and Methods

### 2.1. Study Design

This study involved the analysis of population-based, anonymized data sourced from the Australian Cancer Database for persons aged 20 years or older at diagnosis with cancer. With the exception of basal and squamous cell skin carcinomas, cancer is legislated as a notifiable disease in Australia and must be reported to a cancer registry in the relevant Australian state or territory. Tumors that are benign, borderline malignancy, or in situ are not included. The state and territory registries supply an agreed minimum subset of annual data to the Australian Institute of Health and Welfare [15], which enters these data into the Australian Cancer Database. Collection of the Australian cancer data began on 1 January 1982, and at the time data was requested for this study (December 2018), data entry was complete for all Australian states and territories to 31 December 2014. The data held by the Australian Cancer Database are described in detail in a publicly available Quality Statement [16].

### 2.2. Case Definitions

Cases selected for this study were registered in the Australian Cancer Database under 35 Morphology Codes of the International Classification of Diseases Oncology, 3rd edition, revision 1 (ICD-O-3.1) [17] that were consistent with the diagnosis of PCNSL, as reported by the Central Brain Tumor Registry of the United States (Table S1) [2]. Given the potential prognostic implications of lymphoma subtype and the high representation of DLBCL in PCNSL [1], cases of DLBCL were distinguished from non-DLBCL. Cases of DLBCL were identified strictly by two Morphology Codes (9680: Malignant lymphoma, large B-cell, diffuse, not otherwise specified (NOS); 9684: Malignant lymphoma, large B-cell, diffuse, immunoblastic, NOS), and broadly with the addition of three Morphology Codes expected to include any other cases of DLBCL, but potentially also other types of lymphoma (9590: Malignant lymphoma, NOS; 9591: Malignant lymphoma, non-Hodgkin, NOS; 9675: Malignant lymphoma, mixed small and large cell, diffuse (obsolete)). Cases of lymphoma that had been identified by these criteria were filtered for 22 ICD-O-3.1 Topography Codes corresponding to the CNS, i.e., brain, spinal cord, cranial nerves, meninges, and other CNS locations, including retina (and thus capturing cases of the primary vitreoretinal subset of PCNSL) (Table S2). Cases of lymphoma that fell outside these 22 Topography Codes were considered to represent non-CNS lymphoma for the purposes of comparing incidence and survival between PCNSL and lymphomas that originated outside the CNS. Cases of non-hematological primary CNS cancers, as defined by relevant Disease Classification Codes of the International Statistical Classification of Diseases and Related Health Problems, 10th revision (ICD-10) [18], were also sourced from the Australian Cancer Database (Table S3), to allow incidence and survival comparisons with PCNSL. Person-related information was obtained for all cases, including sex, age at and year of cancer diagnosis, basis of the diagnosis, vital status, and survival days from diagnosis to death (from any cause) or to 31 December 2014.

### 2.3. Statistical Analyses

Observed cancer incidence rates were calculated with Joinpoint Regression Program, version 4.7.0.0 [19] using annual mid-year population estimates published by the Australian Bureau of Statistics. These rates were age-standardized using the most recent Australian Age Standardized Population [20] or World Standard Population [21,22]. Crude and age-standardized rates were reported per 100,000 person-years, and expressed as a mean across the specified time period with the 95% confidence interval (CI). Where not otherwise stated, incidence rates were reported with reference to the Australian population. Trends in incidence rates were modeled with the Joinpoint Regression Program [23,24]. Joinpoint analysis identifies years when there is a change in trend of the incidence rate, and calculates annual percent change (APC) between those years, and average annual percent change (AAPC) over predefined intervals, with 95% CI. A maximum of five trends—or Joinpoints—were used in this modeling, as recommended by the software manufacturer. Survival was computed in IBM SPSS version 25 as Kaplan–Meier estimates, with log-rank comparison tests applied to assess differences. Survival time was defined as the number of days from diagnosis to death from any cause, or 31 December 2014 in patients still alive on that date. Median days of survival and probabilities of survival up to 5 years with 95% CI were also calculated by Kaplan–Meier statistics. For DLBCL, 5-year relative survival ratios (RSRs) with 95% CI were calculated using cumulative 5-year survival probability estimates for the Australian population generated from 2012–2014 Life Table mortality rates [25], following the method described by Dickman and Adami [26]. Five-year RSRs were limited to persons aged 20–79 years at diagnosis because the required Life Table mortality data were not available for the subgroup aged 80 years and over. For all statistical analyses, a *p*-value less than 0.05 was taken to indicate statistical significance.

### 2.4. Human Research Ethics

Release of data by the Australian Cancer Database was approved by the Australian Institute of Health and Welfare following approvals by the cancer registry data custodian of each Australian state and territory, with protocol reviews by local human ethics committees as appropriate to each jurisdiction. The Southern Adelaide Clinical Human Research Ethics Committee, and the New South Wales Population and Health Services Research Ethics Committee conducted human research ethics reviews for the states of South Australia and New South Wales, respectively (approval numbers: HREC/19/SAC/63; 2019/ETH02728), and the Australian Capital Territory Health Research Ethics and Governance Office conducted a governance review for the Australian Capital Territory (approval number: 2019/STE/00087). Approvals of cancer registry data custodians in the states of Victoria, Queensland, Western Australia, and Tasmania, and the Northern Territory did not require additional local human ethics committee review. A waiver of consent was granted by the human research ethics committees that reviewed this research, in accordance with the Australian National Statement on Ethical Conduct in Human Research.

## 3. Results

### 3.1. Patient and Cancer Characteristics in The PCNSL Cohort

A total of 1329 cases of PCNSL were identified in the Australian population between 1982 and 2014, according to the combination of 35 ICD-O-3 Morphology Codes and 22 ICD-O-3.1 Topography Codes used to define this cancer. These cases were registered under 17 of the 35 Morphology Codes (Table S1) and accounted for 1.4% of all lymphoma cases covered by those 17 Morphology Codes (*n =* 93,793). Median age at diagnosis with PCNSL was 66 years (range = 20–100 years), and the cancer was slightly more common in males (*n* = 734, 55.2%). The majority of cases were diagnosed by cytological or histopathological evaluation of tissue samples (*n* = 1230, 92.5%), and the cancer most commonly occurred first in the brain (*n* = 1202, 90.4%), followed by the spinal cord, cranial nerves. and other parts of the CNS including the retina (*n* = 104, 7.8%) and the meninges (*n* = 23, 1.7%). The predominant histopathological type of PCNSL was DLBCL, representing 64.1% of cases with Morphology Codes specifying DLBCL (*n* = 852) and 94.7% of cases with Morphology Codes consistent with DLBCL (*n* = 1259). Less than 2% of PCNSL were T-cell or NK-cell lymphomas. Characteristics of patients and cancers in the PCNSL cohort are presented in Table 1.

### 3.2. Incidence Rates and Trends for PCNSL, Non-CNS Lymphoma and Non-Hematological Primary CNS Cancers

**Figure 1:**
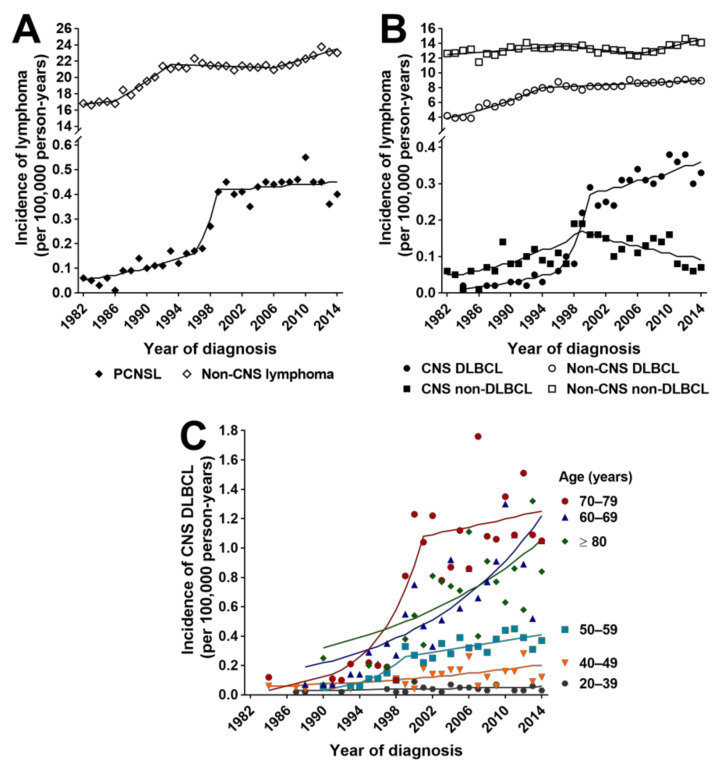
Age-standardized incidence rates over time for Australian adults with (**A**) primary central nervous system lymphoma (PCNSL) and non-CNS lymphoma; (**B**) diffuse large B-cell lymphoma (DLBCL) and other lymphomas (non-DLBCL) within the CNS and non-CNS compartments; (**C**) CNS DLBCL, presented by age group at diagnosis. Shapes indicate observed incidence rates, and continuous lines indicate modeled incidence rates.

Incidence rates of PCNSL and non-CNS lymphoma (identified by 17 ICD-O-3 Morphology Codes) were calculated for the Australian adult population. Age-standardized incidence of PCNSL across the 33-year study period averaged 0.27 (0.21–0.33) per 100,000 person-years, which was considerably lower than the incidence of non-CNS lymphoma (20.58 (19.87–21.30) per 100,000, *n* = 92,464 cases) and the incidence of non-hematological primary CNS cancers (9.03 (8.88–9.17) per 100,000, *n* = 40,326 cases) (Table 2). Trend modeling demonstrated increasing incidence of PCNSL and, by a lesser amount, non-CNS lymphoma during the study period (AAPC = 6.8% (1.7–12.2) and 1.1% (0.7–1.5), respectively) (Table 2 and Figure 1A). However, for PCNSL, there was a short period of particularly rapid increase in incidence from 1996 to 1999, when the incidence of non-CNS lymphoma remained stable. Given the rise in incidence, the study period was divided into two time intervals to permit analysis of temporal trends: 1982–1999 and 2000–2014. Between 2000 and 2014, age-standardized incidence of PCNSL plateaued at a mean of 0.43 (0.41–0.46) per 100,000 person-years; this rate was approximately 50-times less than that of non-CNS lymphoma (21.89 (21.41–22.38) per 100,000). Incidence of non-hematological CNS cancers remained unchanged throughout the study interval. Comparable trends were modeled when cancer cohorts were age-standardized to the world population (Table 2).

### 3.3. Incidence Rates and Trends for CNS and Non-CNS DLBCL

Differences in incidence rates and trends between PCNSL and non-CNS lymphoma were further explored by dichotomizing for DLBCL and non-DLBCL types. Mean age-standardized incidence of CNS DLBCL between 1982 and 2014 was 0.16 (0.11–0.22) per 100,000 person-years (*n* = 852 cases), versus 7.39 (6.80–7.98) per 100,000 for non-CNS DLBCL (*n* = 33,997 cases) (Table 2). Trend modeling indicated an 11.3% (5.7–17.2) growth in mean annual incidence of CNS DLBCL over this period; the annual incidence increased by 12.7% (2.4–24.1) from 1984 to 1996, accelerated to 45.3% (9.0–93.8) between 1996 and 2000, and reduced considerably to 2.1% (0.4–3.8) from 2000 to 2014 (Table 2 and Figure 1B). Eighty-nine percent of all diagnoses of CNS DLBCL were made between 2000 and 2014 (*n* = 759 cases), involving an average of 0.31 (0.29–0.33) per 100,000 adults annually. From 1982 to 2014, the incidence of non-CNS DLBCL increased annually by an average 2.7% (2.2–3.1), with an annual growth prior to 1994 of 6.2% (5.1–7.4) that subsequently stabilized at 0.6% (0.3–0.9) (Table 2 and Figure 1B). Between 2000 and 2014, non-CNS DLBCL affected an average of 8.60 (8.41–8.80) per 100,000 adults annually (*n* = 20,934 cases). In contrast to the trends in incidence of CNS DLBCL, the incidence of CNS non-DLBCL (*n* = 477 cases) increased annually by 7.9% (3.8–12.3) between 1982 and 1999, and decreased annually by 3.9% (−7.0–−0.7) from 1999 to 2014, resulting in no net change in incidence rates over time. The incidence of non-CNS non-DLBCL (*n* = 58,467 cases) also remained relatively constant, growing by just 0.5% (0.1–0.9) annually over the study period (Table 2). From 2000, the increase in incidence of CNS DLBCL was greater than could be accounted for by population growth, as indicated by crude incidence rates (Table S4). When incidence trends for DLBCL were standardized to the world population, similar results were obtained (Table 2). These observations indicate that changes in PCNSL incidence over the study period were driven largely by changes in incidence of DLBCL. Mean observed crude incidence rate of CNS DLBCL—as well as all PCNSL—was higher in males than females, albeit marginally, and trend modeling of crude rates indicated a stable annual rate in males, but suggested growth in the annual rate in females (AAPC = 2.4% (0.0–4.8)) (Table S4).

The impact of age on incidence rates and trends of CNS and non-CNS DLBCL was examined. The increase in annual age-standardized incidence of CNS DLBCL between 1996 and 2000 was greatest in persons aged 70–79 years and 50–59 years at cancer diagnosis (AAPC = 22.9% (11.2–35.9) and 19.6% (9.8–30.3), respectively), but from 2000, this growth rate reduced substantially to an average of 3.0% or less in these age groups (Table 3 and Figure 1C). In those persons aged 60–69 years, annual incidence of CNS DLBCL increased consistently across the study period by 7.5% (4.8–10.2), and thus between 2000 and 2014, annual CNS DLBCL incidence grew most rapidly in that age group, followed by those aged 80 years and over (AAPC = 5.1% (1.8–8.6)). Amongst persons aged 40–49 years, there was also annual growth in incidence during the same period, measured at 4.4% (1.0–7.8). After 2000, the age-standardized incidence of CNS DLBCL was highest in persons aged 70–79 years (1.14 (1.00–1.28) per 100,000 person-years), but with a stable rate of growth. In comparison to CNS DLBCL, annual incidence rates of non-CNS DLBCL increased over differing periods up to 1997 in all age groups from 50 years of age and over (APC = 3.6% (1.0–6.2) to 6.7% (4.7–8.7)), after which the growth in these rates reduced (Figure S1). From 2000 to 2014 specifically, annual incidence of non-CNS DLBCL in these groups plateaued or increased only marginally by an average of 1.3% (range = 0.3% (−0.3–0.9) to 1.3 (0.9–1.7)). Growth in the incidence of non-CNS DLBCL in those aged 40–49 years did not change during this period, and incidence growth rate was unchanged over time in adults aged 20–39 years for both CNS and non-CNS DLBCL (Table 3, Figure 1C and Figure S1). These results indicate that during the study period, increasing age was associated with increasing incidence of DLBCL, although rates of growth over time differed with age group. Incidence of CNS DLBCL increased more rapidly, and increasingly affected younger persons, compared to non-CNS DLBCL.

### 3.4. Survival Outcomes for PCNSL, Non-CNS Lymphoma and Non-Hematological Primary CNS Cancers

**Figure 2:**
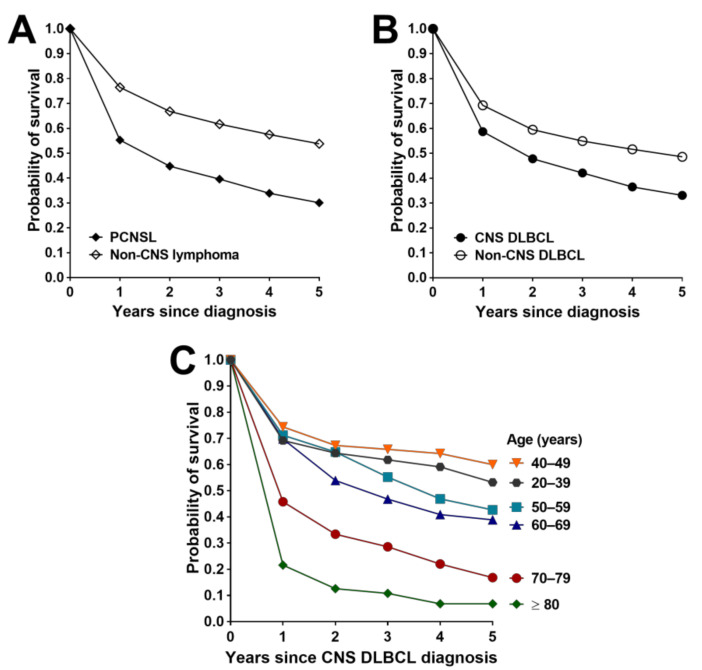
Probability of survival for up to 5 years in Australian adults with (**A**) primary central nervous system lymphoma (PCNSL) and non-CNS lymphoma; (**B**) diffuse large B-cell lymphoma (DLBCL) within the CNS and non-CNS compartments; (**C**) CNS DLBCL presented by age group at diagnosis.

Survival outcomes following a diagnosis of PCNSL or non-CNS lymphoma were determined for the entire study period (1982–2014), as well as for the 1982–1999 and 2000–2014 time intervals in order to identify any temporal changes given recent therapeutic advances. Shorter time intervals were not interrogated due to small and variable case numbers, particularly prior to 2000. Kaplan–Meier log-rank comparisons showed that between 1982 and 2014, survival of persons with PCNSL was significantly lower (*p* < 0.0001) than that of persons with non-CNS lymphoma, for all causes of death (Table 4). Probability of survival at 5 years was 0.30 (0.27–0.33) versus 0.54 (0.53–0.54), and median survival time was 522 days (424–620) versus 2263 days (2185–2267) for individuals with CNS and non-CNS lymphoma, respectively (Table 4 and Figure 2A). Survival after a diagnosis of PCNSL improved significantly between 1982–1999 and 2000–2014 (*p* < 0.001) (Table 4). There was no difference in survival between men and women across the entire study period, including for the two defined time intervals (Table S5). The 5-year survival probability for all persons was 0.21 (0.16–0.26) versus 0.33 (0.30–0.36), and median survival time increased from 318 days (200–436) to 600 days (465–734) in 1982–1999 and 2000–2014, respectively. An approximate doubling in median survival time between these intervals was also observed for non-CNS lymphoma. Survival following diagnosis of a non-hematological CNS cancer improved marginally and was worse than that of PCNSL (Table 4).

### 3.5. Survival Outcomes for CNS and Non-CNS DLBCL

Differences in survival outcomes for PCNSL and non-CNS lymphoma were further evaluated by comparing DLBCL and non-DLBCL types. Survival following a diagnosis of CNS DLBCL was significantly lower (*p* < 0.0001) than that following a diagnosis of non-CNS DLBCL over the 33-year study period, considering death from any cause, with 5-year survival probabilities of 0.33 (0.30–0.37) and 0.49 (0.48–0.49), and median survival times of 639 days (482–796) and 1643 days (1533–1667) for CNS and non-CNS lymphoma, respectively (Table 4). Considering all PCNSL subtypes, the 5-year survival probability for persons with DLBCL was significantly higher than that for persons with non-DLBCL (0.33 (0.30–0.37) versus 0.25 (0.21–0.29)) (Figure 2B). Conversely, for individuals diagnosed with non-CNS lymphoma, 5-year survival for DLBCL was significantly lower than that for non-DLBCL (0.49 (0.48–0.49) versus 0.57 (0.57–0.57)). Between 1982–1999 and 2000–2014, survival after diagnosis of either CNS or non-CNS DLBCL improved significantly (*p* ≤ 0.043), but with less improvement in median survival times for the CNS cancers (from 461 days (297–625) to 690 days (500–880)) versus the non-CNS cancers (from 674 days (632–716) to 2679 days (2565–2792)) (Table 4). There were some differences in survival outcomes for persons with CNS DLBCL by age group at diagnosis: probability of survival at 5 years was highest in age groups under 70 years (range = 0.60 (0.48–0.72) to 0.39 (0.32–0.46)) and markedly lower in age groups of 70 years and older (range = 0.17 (0.11–0.22) to 0.07 (0.01–0.13)), and this observation also applied to persons with non-CNS DLBCL (Figure 2C, Figure S2 and Table S6). Five-year RSRs for those diagnosed with CNS DLBCL indicated an increasing mortality rate associated with the lymphoma with advancing age, ranging from 38–45% for persons diagnosed before 50 years (RSR = 0.54 (0.41–0.70) to 0.62 (0.51–0.74)) versus 75% for persons diagnosed at or after 70 years (RSR = 0.25 (0.19–0.33)) (Table S7). For non-CNS DLBCL, excess mortality by 5 years was also highest in older adults, but considerably lower than that estimated for CNS DLBCL, at 42% for persons diagnosed at or after 70 years (RSR = 0.58 (0.57–0.60)).

## 4. Discussion

This is the first survey of the incidence of PCNSL and the survival of persons with PCNSL in the Australian population. To our knowledge, this is also the first national population-based report that compares these indices with those for lymphoma outside the CNS, and for DLBCL versus non-DLBCL types. Over a 33-year period spanning 1982 to 2014, the incidence of PCNSL in Australia has quadrupled, outpacing the growth in lymphoma incidence outside the CNS. Despite increasing incidence, lymphoma is rare in the CNS, with data from 2000 to 2014 indicating that an average of 4 in every million adults are diagnosed each year, compared to outside the CNS, with 50-times more adults or almost 220 per million cases diagnosed annually. Non-hematological CNS cancers have consistently affected 90 adults per million per year. Changes in PCNSL incidence rates have been associated with changes in incidence of the DLBCL subtype. Despite improvement over the study period, survival outcomes for patients diagnosed with PCNSL remain poor, with a median survival time of less than 2 years and a 5-year survival of 33%. For those persons with lymphoma presenting outside the CNS, expectation of survival is substantially better: median survival is approximately 9 years, and 5-year survival is 62%. The 5-year mortality for CNS DLBCL increases with advancing age, and is greater than that for non-CNS DLBCL. Thus, for persons aged 70–79 years at diagnosis, the excess 5-year mortality rate of CNS DLBCL is nearly double that of non-CNS DLBCL at 75% versus 42%, respectively.

Present incidence of PCNSL in the Australian population is greater than that reported for Korea (i.e., 0.25 per 100,000 person-years) [10], but consistent with figures reported in Japanese, Spanish, French, Irish, Swedish, Dutch, and North American population studies, which range between 0.4 and 0.5 per 100,000 person-years [2,11,12,13,27,28,29]. While reports from Western Europe and Asia indicate ongoing annual increases [9,10,11,12,13,27], incidence of PCNSL has plateaued in the United States [30]. The overall increase in incidence of PCNSL in Australia that we have observed, was characterized by a rapid ‘jump’ in the rate in the 1990s, which was most apparent for persons aged 70–79 years, after which the rate plateaued. This pattern somewhat mirrored, yet lagged behind, that observed in the United States; according to United States population data spanning an overlapping calendar period, there was a similar rapid increase in rate during the 1980s, followed by a relative plateau [30]. The incidence of PCNSL is higher in men than women in Australia, consistent with observations from other population-based cohorts [2,10,12].

Currently in Australia, 72.5% of PCNSL are classified as DLBCL. Studies from Western Europe and North America have reported prevalence rates of CNS DLBCL that range from 51% to 86% in predominantly immunocompetent populations [3,6,9,12,27,29,31]. Dichotomizing our data by DLBCL and non-DLBCL types showed an effect of the DLBCL diagnosis on PCNSL incidence that was masked when all histopathological types were considered together: although incidence of PCNSL appeared to be plateauing, this figure was rising for DLBCL, while decreasing for non-DLBCL. Indeed, the ‘jump’ in incidence of PCNSL in the 1990s was driven by a rapid increase in CNS DLBCL. The increasing incidence of CNS DLBCL that we observed in persons aged 60 years and over parallels results of other recent population-based studies [10,11,12,30]. However, while these other studies report that incidence rates have remained constant for those aged below 50 years, our data suggest that people aged 40–49 years are also increasingly affected by CNS DLBCL. Interestingly, the incidence of non-Hodgkin lymphoma in Australian men was recently reported to be second highest in the world [32]. At this time, the factors that may be driving the observed increase in CNS DLBCL in Australia remain uncertain. Fluctuations in incidence relative to age groups in Australia for CNS DLBCL do not resemble those for PCNSL reported in the United States for the HIV epidemic of the 1980s and 1990s [7,30,33,34]. The increasing incidence of PCNSL that we report also cannot be explained simply on the basis of an aging population or improved diagnostic and classification tools (including changes in coding usage over time, Figure S3) as also noted elsewhere [9,30,32,35]; particular age groups are affected differently, and there has been no increase in the incidence of non-CNS DLBCL in parallel with that of CNS DLBCL. However, these factors may be contributing to the observed changes [11,13,30,32,33,36].

Considering deaths from all causes, we calculated survival outcomes for patients with PCNSL, including CNS DLBCL, that are consistent with similar longitudinal reports from other high-income countries [10,11,30], including no differences between sexes, worsening prognosis with advancing age at diagnosis, and improvement in survival expectation over time. An increase in overall and 5-year survival, and median days of survival, has been associated with advances in therapeutic interventions [6,30]. Over the past two decades, diagnostic and treatment regimens have been developed to target molecular variations in lymphoma—particularly in DLBCL types—producing clinical benefit [37,38,39,40,41,42,43]. However, as our work highlights, translating therapeutic advancements for control of lymphoma outside the CNS to treatment of disease inside the CNS remains a challenge [44,45]. Blood–brain and blood–tumor barriers limit penetration of drugs into the CNS compartment [46], and divergent cell-of-origin within the DLBCL group impacts responses to therapeutics [36,41]. Our data support the premise that there is prognostic value in considering PCNSL survival outcomes based on histopathological type, specifically DLBCL versus non-DLBCL [6,47,48].

Direct comparisons of incidence rates and survival outcomes for PCNSL using national population datasets have limitations. Importantly, a consensus ICD-O-3.1 Morphology and Topography Code definition of PCNSL, and one which captures the primary vitreoretinal lymphoma subgroup, is lacking. We followed the Morphology Code-based definition used by the Central Brain Tumor Registry of the United States [2], and included optic nerve and retina in our Topography Code-based definition of the CNS. However, divergent PCNSL definitions exist in the literature, and limit comparative analyses between population-based studies [4]. In addition, there are differing levels of population coverage and data collected across registries, and treatment approaches vary between countries [32]. Furthermore, incidence rates need to be considered in light of the population data from which they are calculated, and ideally be standardized to that population. Data on cause of death, synchronous or secondary cancers, immune status and treatment history are not lodged in the Australian Cancer Database, and thus it is not possible to link our observations to factors of predisposition or prognostic determinants beyond age and histopathology.

## 5. Conclusions

Our study provides the first Australian population-based perspective on incidence and survival of PCNSL, with emphasis on the DLBCL type, and in comparison to lymphoma outside the CNS. Observations that we have made share some similarities, but also differences, with those reported for a range of culturally and ethnically diverse populations from other high-income countries. Results of our study suggest there would be value in an international effort to develop a consensus framework and set of definitions for the interrogation of national datasets that describe the epidemiology of PCNSL.

## Figures and Tables

**Table 1:** Characteristics of the PCNSL cohort sourced from the Australian Cancer Database. *n* = 1329 persons.

Characteristics	N (%) or Median (Range)
Median age at diagnosis (years)	66 (20–100)
Sex	‒
Males	734 (55.2)
Females	595 (44.8)
Basis of diagnosis	‒
Histopathology	1189 (89.4)
Cytology	41 (3.1)
Clinical diagnosis without tissue specimen	86 (6.5)
Other ^§^	13 (1.0)
Prevalent types: ICD-O-3.1 Morphology Code	‒
DLBCL	‒
Strict definition: 9680, 9684	852 (64.1)
Broad definition: 9590, 9591, 9675, 9680, 9684	1259 (94.7)
T-cell, NK-cell lymphoma: 9702, 9714, 9719	24 (1.8)
Other: 9590	239 (17.9)
Distribution by initial site of CNS involvement	‒
Brain	1202 (90.4)
Meninges	23 (1.7)
Spinal cord, cranial nerve, other	104 (7.8)

Abbreviations: CNS, central nervous system; PCNSL, primary central nervous system lymphoma; DLBCL, diffuse large B-cell lymphoma; N, total number. ^§^ Death certificate or unknown.

**Table 2:** Age-standardized incidence rates and trends for CNS and non-CNS lymphomas, and non-hematological CNS cancers in Australian adults between 1982 and 2014.

Cancer Subset	Age-Standardized ^§^ Incidence Per 100,000 Person-Years							
	Australian Standard Population				World Standard Population			
	Observed Mean (95% CI)		AAPC (%) (95% CI)		Observed Mean (95% CI)		AAPC (%) (95% CI)	
	1982–2014	2000–2014	1982–2014	2000–2014	1982–2014	2000–2014	1982–2014	2000–2014
PCNSL	0.27 (0.21–0.33)	0.43 (0.41–0.46)	6.8 (1.7–12.2) *	0.5 (−0.9–1.9)	0.23 (0.18–0.28)	0.36 (0.34–0.39)	6.5 (3.0–10.1) *	−0.1 (−1.7–1.5)
Non-CNS lymphoma	20.58 (19.87–21.30)	21.89 (21.41–22.38)	1.1 (0.7–1.5) *	0.7 (0.4–1) *	16.91 (16.36–17.46)	17.88 (17.48–18.27)	1.0 (0.6–1.4) *	0.7 (0.4–1) *
CNS DLBCL	0.16 (0.11–0.22)	0.31 (0.2–0.33)	11.3 (5.7–17.2) *^,^^‡^	2.1 (0.4–3.8) *	0.14 (0.09–0.18)	0.26 (0.24–0.28)	10.1 (4.6–15.9) *^,^^‡^	2.1 (0.4–3.9) *
Non-CNS DLBCL	7.39 (6.80–7.98)	8.60 (8.4–8.80)	2.7 (2.2–3.1) *	0.6 (0.3–0.9) *	6.03 (5.56–6.49)	6.94 (6.81–7.08)	2.7 (2.2–3.1) *	0.5 (0.2–0.8) *
CNS non-DLBCL	0.10 (0.09–0.12)	0.12 (0.10–0.14)	2.2 (−0.3–4.8)	−3.9 (−7–−0.7) *	0.09 (0.08–0.10)	0.10 (0.08–0.12)	1.5 (−0.9–3.9)	−4.8 (−8.2–−1.3) *
Non-CNS non-DLBCL	13.19 (12.97–13.42)	13.29 (12.91–13.66)	0.5 (0.1–0.9) *	0.6 (0–1.3) *	10.88 (10.70–11.06)	10.93 (10.61–11.25)	0.4 (0–0.8) *	0.7 (0.1–1.4) *
Non-hematological CNS cancers	9.03 (8.88–9.17)	9.26 (9.07–9.45)	0.0 (−0.3–0.3)	−0.5 (−1.2–0.3)	7.96 (7.86–8.06)	8.09 (7.93–8.25)	−0.1 (−0.4–0.3)	−0.5 (−1.2–0.2)

Abbreviations: PCNSL, primary central nervous system lymphoma; CNS, central nervous system; DLBCL, diffuse large B-cell lymphoma; AAPC, average annual percent change; CI, confidence interval. ^§^ Age-standardized using the “Australian Bureau of Statistics Standard Population 2001” or “World Health Organization World Standard Population 2000–2025” for all persons aged ≥20 years. * JoinPoint trend significance, *p* ≤ 0.05. ^‡^ Modeled from first case registered in 1984.

**Table 3:** Incidence rates and trends for CNS DLBCL in Australian adults by age group at diagnosis between 2000 and 2014.

Age (Years)	Age-Standardized ^§^ Incidence Per 100,000 Person-Years	
	Observed Mean (95% CI)	AAPC (%) (95% CI)
20–29	0.03 (0.01–0.05)	–0.1 (-5.3–5.5)
30–39	0.06 (0.04–0.08)	2.1 (−1.9–6.4)
40–49	0.14 (0.10–0.18)	4.4 (1.0–7.8) *
50–59	0.34 (0.29–0.37)	3.0 (1.0–5.1) *
60–69	0.77 (0.63–0.92)	7.5 (4.8–10.2) *
70–79	1.14 (1.00–1.28)	2.6 (−1.1–6.4)
≥80	0.76 (0.62–0.89)	5.1 (1.8–8.6) *

Abbreviations: CNS, central nervous system; DLBCL, diffuse large B-cell lymphoma; AAPC, average annual percent change; CI, confidence interval. ^§^ Age-standardized using the “Australian Bureau of Statistics Standard Population 2001”. * JoinPoint trend analysis significance, *p* < 0.05.

**Table 4:** Survival outcomes for CNS and non-CNS lymphomas, and non-hematological CNS cancers in Australian adults between 1982 and 2014.

Cancer Subset	Time Interval									
	1982–2014			1982–1999			2000–2014			*p*-Value *
	N	Median Survival (Days) (95% CI)	5-Year Survival (Probability) (95% CI)	N	Median Survival (Days) (95% CI)	5-Year Survival (Probability) (95% CI)	N	Median Survival (Days) (95% CI)	5-Year Survival (Probability) (95% CI)	Overall Survival
PCNSL	1329	522 (424–620)	0.30 (0.27–0.33)	283	318 (200–436)	0.21 (0.16–0.26)	1046	600 (465–734)	0.33 (0.30–0.36)	<0.001
Non-CNS lymphoma	92,464	2263 (2185–2267)	0.54 (0.53–0.54)	39,193	1318 (1278–1358)	0.45 (0.44–0.45)	53,271	3388 (3310–3466)	0.62 (0.62–0.62)	<0.001
*p*-value ^§^	<0.0001	NA	NA	<0.0001	NA	NA	<0.0001	NA	NA	NA
CNS DLBCL	852	639 (482–796)	0.33 (0.30–0.37)	93	461 (297–625)	0.24 (0.15–0.32)	759	690 (500–880)	0.34 (0.31–0.38)	0.043
Non-CNS DLBCL	33,997	1643 (1533–1667)	0.49 (0.48–0.49)	13,063	674 (632–716)	0.38 (0.37–0.38)	20,934	2679 (2565–2792)	0.56 (0.56–0.57)	<0.001
*p*-value ^§^	<0.0001	NA	NA	<0.0001	NA	NA	<0.0001	NA	NA	NA
CNS non-DLBCL	477	337 (227–447)	0.25 (0.21–0.29)	190	244 (85–403)	0.20 (0.14–0.25)	287	425 (271–579)	0.30 (0.24–0.35)	0.01
Non-CNS non-DLBCL	58,467	2553 (2500–2606)	0.57 (0.57–0.57)	26,130	1640 (1588–1692)	0.48 (0.47–0.48)	32,337	3773 (3666–3880)	0.66 (0.65–0.66)	<0.001
*p*-value ^§^	<0.0001	NA	NA	<0.0001	NA	NA	<0.0001	NA	NA	NA
Non-hematological CNS cancers	40,326	277 (272–282)	0.19 (0.19–0.20)	18,151	222 (215–229)	0.18 (0.17–0.18)	22,175	329 (321–336)	0.20 (0.20–0.21)	<0.0001

Abbreviations: PCNSL, primary central nervous system lymphoma; CNS, central nervous system; DLBCL, diffuse large B-cell lymphoma; CI, confidence interval; *n*, number of cases; NA, not applicable. ^§^ *p*-value for Kaplan–Meier log-rank pairwise comparisons of probability of survival overall (N) between lymphoma inside versus outside the CNS. * *p*-value for Kaplan–Meier log-rank pairwise comparisons of probability of survival overall (N) for cancers diagnosed in 1982–1999 versus 2000–2014.

## Data Availability

Third-party data. Restrictions apply on the availability of these data. Data was obtained from the Australian Cancer Database held by the Australian Institute of Health and Welfare (AIHW) and are available via https://www.aihw.gov.au/or-services/data-on-request with the permission of the AIHW.

## Supplementary Materials

The following are available online at https://www.mdpi.com/2072-6694/13/3/403/s1, Table S1: ICD-O-3.1 Morphology Codes used to identify PCNSL cases in the Australian Cancer Database (1982 to 2014), Table S2: ICD-O-3.1 Topography Codes used to identify PCNSL cases in the Australian Cancer Database (1982 to 2014), Table S3: ICD-10 Disease Classification Codes used to identify non-hematological CNS cancers in the Australian Cancer Database (1982 to 2014), Table S4: Crude incidence rates and trends for PCNSL and CNS DLBCL in Australian adults by sex between 2000 and 2014, Table S5: Survival outcomes for PCNSL in Australian adults by sex between 1982 and 2014, Table S6: Survival outcomes for CNS and non-CNS DLBCL by age groups at diagnosis between 1982 and 2014, Table S7: Five-year relative survival ratios for CNS and non-CNS DLBCL by age group at diagnosis between 1982 and 2014, Figure S1: Age-standardized incidence rates over time for Australian adults with noncentral nervous system (CNS) diffuse large B-cell lymphoma (DLBCL), presented by age group at diagnosis. Figure S2: Kaplan–Meier probability of survival for up to 5 years in Australian adults with noncentral nervous system (CNS) diffuse large B-cell lymphoma (DLBCL), presented by age group at diagnosis. Figure S3: Frequency of ICD-O-3.1 Morphology Codes (International Classification of Diseases Oncology, third edition, revision 1) indicating diffuse large B-cell (DLBCL) versus non-DLBCL in Australian adults identified with primary central nervous system lymphoma (PCNSL) in 1982–1999 (black bars) and 2000–2014 (grey bars).
