# Supplementary material for: Changing Incidence and Survival of Primary Central Nervous System Lymphoma in Australia: A 33-Year National Population-Based Study
Source: Cancers (Basel). 2021 Jan 22;13(3):403. doi: 10.3390/cancers13030403 (PMC7865336; doi:10.3390/cancers13030403)
Supplement: Supplementary file 1 [file cancers-13-00403-s001.pdf]

cancers

MDPI

Article

Changing Incidence and Survival of Primary Central Nervous System Lymphoma in Australia: A 33-Year National Population-Based Study

Alexandra L. Farrall and Justine R. Smith

Supplementary Materials

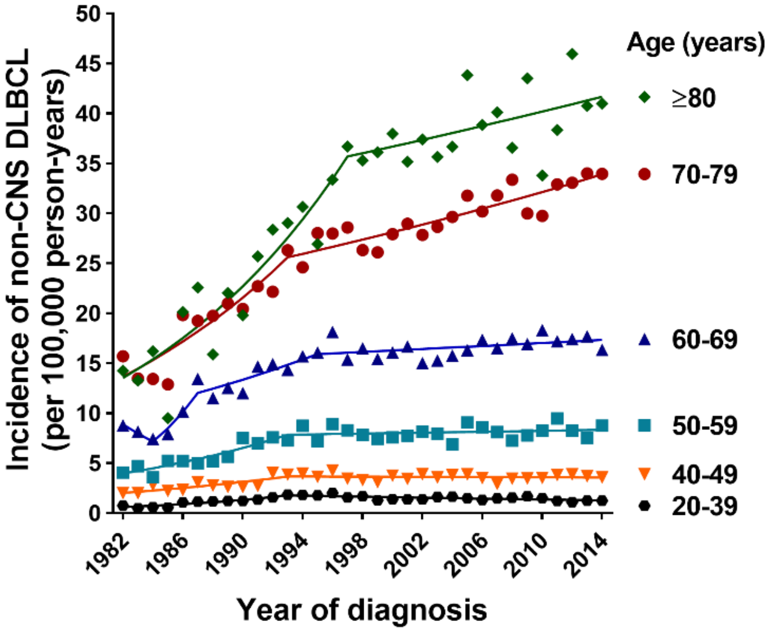

Figure S1. Age-standardized incidence rates over time for Australian adults with non-central nervous system (CNS) diffuse large B-cell lymphoma (DLBCL), presented by age group at diagnosis. Shapes indicate observed incidence rates, and continuous lines indicate modelled incidence rate.

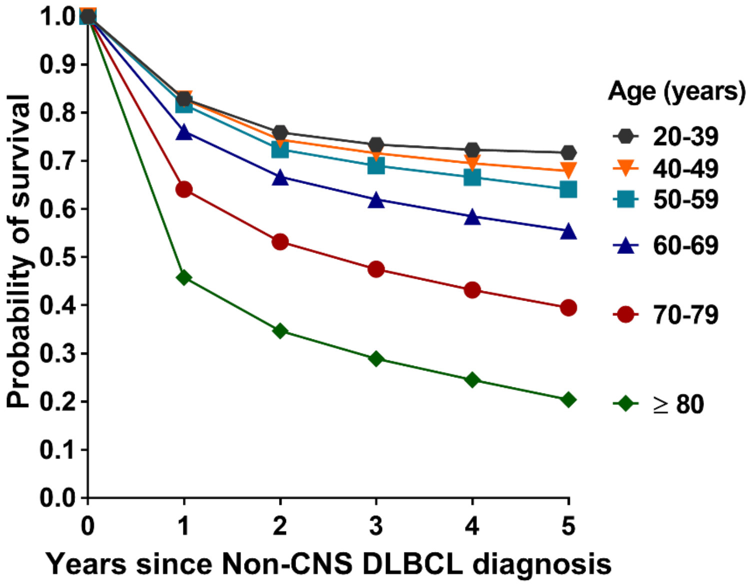

Figure S2. Kaplan-Meier probability of survival for up to 5 years in Australian adults with non-central nervous system (CNS) diffuse large B-cell lymphoma (DLBCL), presented by age group at diagnosis.

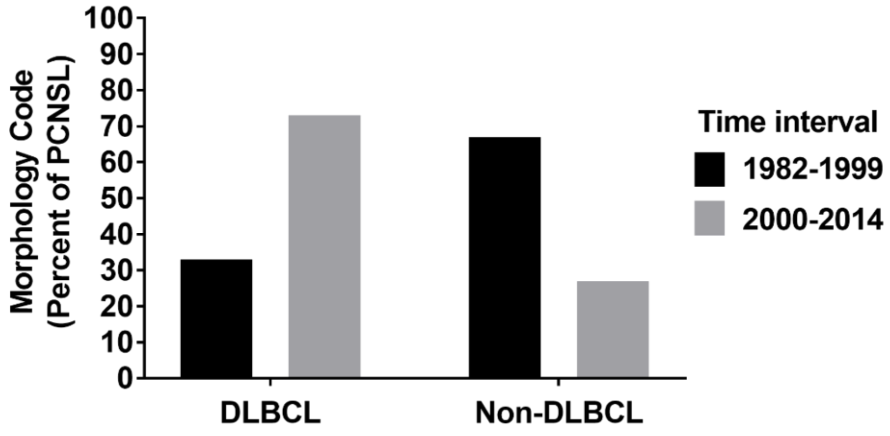

Figure S3. Frequency of ICD-O-3.1 Morphology Codes (International Classification of Diseases Oncology, third edition, revision 1) indicating diffuse large B-cell (DLBCL) versus non-DLBCL in Australian adults identified with primary central nervous system lymphoma (PCNSL) in 1982-1999 (black bars) and 2000-2014 (grey bars).

Table S1. ICD-O-3.1 Morphology Codes used to identify PCNSL cases in the Australian Cancer Database (1982 to 2014), following the methods of Ostrom QT et al. [2].

Morphology Code	Term Description	Cases in Australian Cancer Database (N)
	**959–972 Hodgkin and non-Hodgkin lymphomas**	–
**9590**	**Malignant lymphoma, NOS**	**239**
**9591**	**Malignant lymphoma, non-Hodgkin, NOS**	**165**
9596	Composite Hodgkin and non-Hodgkin lymphoma	–
**9650**	**Hodgkin lymphoma, NOS**	**1**
9651	Hodgkin lymphoma, lymphocyte-rich	–
9652	Hodgkin lymphoma, mixed cellularity, NOS	–
**9653**	**Hodgkin lymphoma, lymphocyte depletion, NOS**	**1**
9654	Hodgkin lymphoma, lymphocyte depletion, diffuse fibrosis	–
9655	Hodgkin lymphoma, lymphocyte depletion, reticular	–
9659	Hodgkin lymphoma, nodular lymphocyte predominance	–
9661	Hodgkin granuloma [obs]	–
9662	Hodgkin sarcoma [obs]	–
9663	Hodgkin lymphoma, nodular sclerosis, NOS	–
9664	Hodgkin lymphoma, nodular sclerosis, cellular phase	–
9665	Hodgkin lymphoma, nodular sclerosis, grade 1	–
9667	Hodgkin lymphoma, nodular sclerosis, grade 2	–
**9670**	**Malignant lymphoma, small B lymphocytic, NOS**	**19**
**9671**	**Malignant lymphoma, lymphoplasmacytic**	**4**
9673	Mantle cell lymphoma	–
**9675**	**Malignant lymphoma, mixed small and large cell, diffuse [obs]**	**3**
**9680**	**Malignant lymphoma, large B-cell, diffuse, NOS**	**817**
**9684**	**Malignant lymphoma, large B-cell, diffuse, immunoblastic, NOS**	**35**
**9687**	**Burkitt lymphoma, NOS**	**7**
9690	Follicular lymphoma, NOS	–
**9691**	**Follicular lymphoma, grade 2**	**7**
**9695**	**Follicular lymphoma, grade 1**	**2**
**9698**	**Follicular lymphoma, grade 3**	**2**
**9699**	**Marginal zone B-cell lymphoma, NOS (MALT)**	**3**
	**970–971 Mature T- and NK-cell lymphomas**	–
9701	Sezary syndrome	–
**9702**	**Mature T-cell lymphoma, NOS**	**18**
9705	Angioimmunoblastic T-cell lymphoma	–
**9714**	**Anaplastic large cell lymphoma, T cell and Null cell type**	**2**
**9719**	**NK/T-cell lymphoma, nasal and nasal-type**	**4**
	**972 Precursor cell lymphoblastic lymphoma**	–
9728	Precursor B-cell lymphoblastic lymphoma	–
9729	Precursor T-cell lymphoblastic lymphoma	–
	**TOTAL**	**1329**

Abbreviations: ICD-O-3.1, International Classification of Diseases Oncology, third edition, revision 1; PCNSL, primary central nervous system lymphoma; NOS, not otherwise specified; [obs], obsolete.
Lymphoma Morphology Codes identified in the CNS from the Australian Cancer Database (N) are highlighted in bold text.

Table S2. ICD-O-3.1 Topography Codes used to identify PCNSL cases in the Australian Cancer Database (1982 to 2014).

Topography Codes	Sites
C70.0, C70.1 and C70.9	Meninges
C71.0, C71.1, C71.2, C71.3, C71.4, C71.5, C71.6, C71.7, C71.8 and C71.9	Brain
C72.0, C72.1, C72.2, C72.3, C72.4, C72.5, C72.8 and C72.9	Spinal cord, cranial nerves, and other parts of the central nervous system (including optic nerve C72.3)
C69.2	Retina

Abbreviations: ICD-O-3.1, International Classification of Diseases Oncology, third edition, revision 1; PCNSL, primary central nervous system lymphoma.

Table S3. ICD-10 Disease Classification Codes used to identify non-hematological CNS cancers in the Australian Cancer Database (1982 to 2014).

Disease Classification Codes	Term Description
C70.0 to C70.9	Malignant neoplasms of the meninges
C71.0 to C71.9	Malignant neoplasms of the brain (Excluding cranial nerves (C72.2-C72.5) and retrobulbar tissue (C69.6)).
C72.0 to C72.9	Malignant neoplasm of the spinal cord, cranial nerves, and other parts of the central nervous system (Excluding meninges (C70._) and peripheral nerves and autonomic nervous system (C47._))
C69.2	Retina

Abbreviations: ICD-10, International Classification of Diseases and Related Health Problems, 10th revision (version 2010); CNS, central nervous system.

Table S4. Crude incidence rates and trends for PCNSL and CNS DLBCL in Australian adults by sex between 2000 and 2014.

Sex	N	Crude incidence § per 100,000 person-years	
		Observed mean (95% CI)	AAPC (95% CI)
PCNSL			
All	1046	0.45 (0.42–0.48)	1.1 (−0.3–2.5)
Males	570	0.50 (0.47–0.52)	1.1 (−0.8–3.1)
Females	476	0.40 (0.35–0.45)	2.3 (−0.4–5.0)
CNS DLBCL			
All	759	0.32 (0.29–0.35)	2.7 (1.0–4.4) *
Males	398	0.35 (0.31–0.38)	1.6 (−2.8–62)
Females	361	0.30 (0.27–0.34)	2.4 (0.0–4.8) *

Abbreviations: PCNSL, primary central nervous system lymphoma; CNS, central nervous system; DLBCL, diffuse large B-cell lymphoma; N, total number; AAPC, average annual percent change; CI, confidence interval; NA, not applicable.

§ Crude incidence was calculated using the annual mid-year population size according to the "Australian Bureau of Statistics" for all persons aged ≥20 years. * JoinPoint trend significance, $p \leq 0.05$.

Table S5. Survival outcomes for PCNSL in Australian adults by sex between 1982 and 2014.

Sex	N	5-Year Survival (Probability) (95% CI)	Median Survival (Days) (95% CI)
Males	734	0.29 (0.26–0.33)	452 (335–569)
Females	595	0.31 (0.27–0.35)	578 (409–747)
p-value§	NS *	NA	NA

Abbreviations: PCNSL, primary central nervous system lymphoma; N, total number; CI, confidence interval; NA, not applicable; NS, not significant. § *p*-value for Kaplan-Meier log-rank pairwise comparisons of probability of survival overall (N). * There were no significant differences in survival outcomes between sexes within or across the 1982–1999 and 2000–2014 time periods.

Table S6. Survival outcomes for CNS and non-CNS DLBCL by age group at diagnosis between 1982 and 2014.

Age (Years)	CNS DLBCL			Non-CNS DLBCL			*p*-Value §
	N	5-Year Survival (Probability) (95% CI)	Median Survival (Days) (95% CI)	N	5-Year Survival (Probability) (95% CI)	Median Survival (Days) (95% CI)	
20–39	46	0.53 (0.38–0.69)	2332 (112–4551)	2510	0.72 (0.70–0.74)	not reached	0.001
40–49	76	0.60 (0.48–0.72)	3231 (1975–4487)	2895	0.68 (0.66–0.70)	7963 (6922–9004)	0.001
50–59	155	0.43 (0.34–0.52)	1280 (859–1701)	5232	0.64 (0.63–0.65)	4634 (4287–4892)	<0.001
60–69	255	0.39 (0.32–0.46)	932 (671–1193)	7925	0.56 (0.54–0.57)	2518 (2365–2671)	<0.001
70–79	231	0.17 (0.11–0.22)	268 (150–386)	9257	0.40 (0.38–0.41)	915 (845–985)	<0.001
≥80	89	0.07 (0.01–0.13)	81 (45–117)	6178	0.20 v0.19–0.22)	296 (277–315)	<0.001
Total	852	0.33 (0.30–0.37)	639 (482–796)	33997	0.49 (0.48–0.49)	1643 (1533–1667)	<0.0001

Abbreviations: CNS, central nervous system; DLBCL, diffuse large B-cell lymphoma; N, total number; CI, confidence interval. § *p*-value for Kaplan-Meier log-rank pairwise comparisons of probability of overall survival.

Table S7. Five-year relative survival ratios for CNS and non-CNS DLBCL by age group at diagnosis between 1982 and 2014.

Age (Years)	5-Year Relative Survival Ratio (Probability) § (95% CI)	
	CNS DLBCL	Non-CNS DLBCL
20–39	0.54 (0.41–0.70)	0.72 (0.71–0.74)
40–49	0.62 (0.51–0.74)	0.70 (0.68–0.71)
50–59	0.45 (0.38–0.54)	0.68 (0.67–0.69)
60–69	0.45 (0.38–0.52)	0.64 (0.63–0.65)
70–79	0.25 (0.19–0.33)	0.58 (0.57–0.60)
≥ 80	ND	ND

Abbreviations: CNS, central nervous system; DLBCL, diffuse large B-cell lymphoma; ND, not determined. § Relative survival ratios were calculated using cumulative probabilities of survival as determined from the "Australian Bureau of Statistics" 2012–2014 Life Table mortality rates for all persons aged 20–79 years.
